# A Biosensor Platform for Point-of-Care SARS-CoV-2 Screening

**DOI:** 10.3390/bios12070487

**Published:** 2022-07-03

**Authors:** Antonios Georgas, Konstantinos Agiannis, Vasiliki Papakosta, Panagiotis Priftis, Spyridon Angelopoulos, Angelo Ferraro, Evangelos Hristoforou

**Affiliations:** School of Electrical and Computer Engineering, National Technical University of Athens, 15780 Athens, Greece; k.agiannis@yandex.com (K.A.); papakostavasiliki@gmail.com (V.P.); priftpan@yahoo.gr (P.P.); spyrosag@central.ntua.gr (S.A.); an.ferraro2@gmail.com (A.F.); hristoforou@ece.ntua.gr (E.H.)

**Keywords:** virus monitoring, SARS-CoV-2, internet of things, screening, readout, smartphone, portable, interdigitated electrodes

## Abstract

The COVID-19 pandemic remains a constant threat to human health, the economy, and social relations. Scientists around the world are constantly looking for new technological tools to deal with the pandemic. Such tools are the rapid virus detection tests, which are constantly evolving and optimizing. This paper presents a biosensor platform for the rapid detection of spike protein both in laboratory conditions and in swab samples from hospitalized patients. It is a continuation and improvement of our previous work and consists of a microcontroller-based readout circuit, which measures the capacitance change generated in an interdigitated electrode transducer by the presence either of sole spike protein or the presence of SARS-CoV-2 particles in swab samples. The circuit efficiency is calibrated by its correlation with the capacitance measurement of an LCR (inductance (L), capacitance (C), and resistance (R)) meter. The test result is made available in less than 2 min through the microcontroller’s LCD (liquid-crystal display) screen, whereas at the same time, the collected data are sent wirelessly to a mobile application interface. The novelty of this research lies in the potential it offers for continuous and effective screening of SARS-CoV-2 patients, which is facilitated and enhanced, providing big data statistics of COVID-19 in terms of space and time. This device can be used by individuals for SARS-CoV-2 testing at home, by health professionals for patient monitoring, and by public health agencies for monitoring the spatio-temporal spread of the virus.

## 1. Introduction

The COVID-19 pandemic has proven to be a major and continuous threat to humanity, as the many mutations and the very fast transmission of the virus lead to the infection of a large number of people. Scientists all over the world are constantly calling for new ways to control the spread of the virus, which includes developing new diagnostic tools for the detection of SARS-CoV-2.

The standard method for virus detection is real-time PCR. Despite its high efficiency, real-time PCR is a time-consuming and costly method, which requires trained personnel and is not available in remote settings. Therefore, it is important to develop reliable devices for point of care (PoC) virus detection [1].

The most common devices used for PoC virus screening are rapid antigen tests, which, however, show much worse performance than real-time PCR [2,3,4]. An explanation for this worse performance can be given by the fact that the rapid antigen tests are based on visual observation of the results, meaning they only provide qualitative results that cannot be automatically processed [5]. Another explanation could be given by the constant mutations of the virus, which cause changes in its structure, such as in the winding domain of the S protein. These changes reduce the effectiveness of rapid tests based on antibody binding, as well as reduce the effectiveness of vaccines [6].

The problem of the visual-only observation and qualitative results could be solved by developing biosensors that can provide an electrical measurement, meaning a faster response time, improved sensitivity, and the possibility of electronic processing of the results [7,8]. The low sensitivity of antigen tests is primarily caused by the visual read-out, because of the intensity of the colored mark that might be difficult to be observed. Additionally, the chemical reaction between the antigen and the ligand plays a role, especially when virus variants arise, changing the affinity between the targeted protein and the ligand [9]. The issue of virus variants affects less the real-time PCR test [10]. Generally, the real-time PCR test takes advantage of genomic regions that are less prone to mutations. Therefore, the real-time PCR test is able to perform properly, even in presence of mutations affecting, e.g., the S protein. Furthermore, since the basic principle of real-time PCR is the amplification of a few copies of the virus’ RNA, the sensitivity of such a test is superior to the antigen test which relays only on the amount of antigen collected from the patient. The above-mentioned examples put the electronic biosensors in a better position since they can utilize all the information contained in the analyte and give quantitative results [11].

Electrochemical biosensors, especially, require simple instrumentation and are highly sensitive, cost-effective, and can be miniaturized. These specifications make them an ideal choice for PoC screening tests [12,13]. The possibility of electronic processing of screening test results means that the spread of the virus in space and time can be controlled. With the use of electronic methods and the Internet of Things (IoT), effective control of the distribution of positive test cases in specific geographical areas, as well as in specific time intervals can be accomplished [14,15]. In fact, by storing the screening results data on a platform, statistical processing can be performed, which may give indications for the improvement of the diagnostic tools themselves, but also for the improvement of the strategy for dealing with the pandemic [16].

Even though the development of various biosensors has been reported [17,18,19,20,21,22], only a few of them have been used as complete SARS-CoV-2 screening devices, especially as complete standalone platforms that perform the diagnosis and electronic processing of the result. Some examples of standalone platforms are the Lucira (San Francisco, CA, USA) [23,24] and Cue (San Diego, CA, USA) [25,26] devices, which can perform amplification of RNA viruses. These two devices can deliver results in about 30 min, which is the minimum time required to perform a PCR test. While the operation of many biosensors can be proven in the laboratory, and achieve quite high levels of sensitivity and efficiency, the biosensors cannot be used in the struggle against the virus until they are tested in the field. There are quite a few challenges to overcome when attempting to convert an electrochemical biosensor to a PoC device, mainly concerning its stability and reproducibility, and its sensitivity to unprocessed real samples [27].

In our previous work, we developed a label-free SARS-CoV-2 electrochemical biosensor based on the binding of the virus structural spike (S) protein to ACE2 protein [28]. ACE2 is immobilized in an interdigitated electrode (IDE) transducer [29], and the binding of the S protein (or the virus through S protein) to ACE2 [30] results in a change in the IDE electrical properties [31], hence its effective capacitance. Up to this point, there are cases of similar biosensors reported, that use ACE2 receptors to bind S protein [32,33,34]. However, while various very interesting biosensors are being developed with excellent results, there are not many reports on their conversion to PoC devices.

This is exactly what we are trying to achieve in this study. After developing the biosensor and validating its operation [28], we developed a prototype electronic readout circuit for the sensor, as well as an Android application that reads the biosensor results remotely through Bluetooth. In this way, a portable microcontroller-based electronic readout circuit was developed, which performs effective capacitance measurements. The screening test results are available on the user’s mobile phone within 2 min, in a friendly to use way. Preliminary results of this work were presented in a conference paper [35]. However, in the continuation of our research, more experiments were conducted, even with real patient samples and not only with S protein, leading to new results and conclusions that optimize the device’s functionality.

## 2. Materials and Methods

### 2.1. Biosensor Preparation

The biosensor preparation procedure has been previously reported [28]. Gold interdigitated electrodes with an electrode length of 7 mm and an electrode surface area of 8.45 mm^2^ were purchased from DropSens (Asturias, Spain). On top of the electrodes, ACE2 protein was immobilized. To verify the functionality of the device, S protein was placed on top of the biosensor, resulting in its binding to ACE2 and therefore a change in the electrical characteristics. Moreover, real virus samples acquired from hospitalized patients were used and the biosensor results for these samples were correlated with real-time PCR results for the same samples. ACE2 and S protein were purchased from InvivoGen (San Diego, CA, USA). All the chemicals used were purchased from Sigma-Aldrich (St. Louis, MO, USA).

### 2.2. Readout Circuit

**Figure 1:**
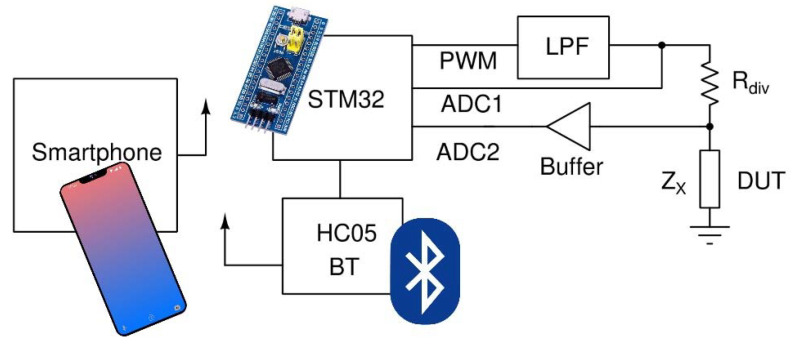
Working principle of the circuit.

In order to integrate the biosensor to a PoC device, the benchtop LCR meter (Hewlett Packard, model 4284A Precision) that was used in the laboratory should be replaced by a precision electronic circuit that is able to measure the capacitance, as well as the resistance of the biosensor. Such a prototype LCR meter was designed and developed. The circuit is able to measure capacitance, ranging from 1 pF up to 3 μF. The main parts of the design are shown in Figure 1.

The design utilizes an STM32 (STM32F103C8T6) microcontroller unit (MCU), able to generate a high-frequency pulse-width modulated (PWM) signal, which is fed to a low pass filter (LPF). The LPF was designed as a second-order Butterworth filter [36] with a cutoff frequency of 13 kHz. The output of the LPF, which was either 1 kHz or 10 kHz sinewave, drove a voltage divider consisting of a known resistor and the device under test (DUT). By measuring the amplitudes of the ADC1 and ADC2 voltages, as well as their phase difference, we could compute the impedance of the DUT based on the following formulas:

(1) $$Re\left(Z_{x}\right)=\frac{R_{div}\left|V_{2}\;\right|\left(V_{1}\;cos\left(\varphi \right)-\left|V_{2}\right|\right)}{V_{1}{}^{2}-2V_{1}\;\left|V_{2}\right|\;cos\left(\varphi \right)+{\left|V_{2}\right|}^{2}}$$

(2) $$Im\left(Z_{x}\right)=\frac{V_{1}R_{div}\left|V_{2}\;\right|sin\left(\varphi \right)}{V_{1}{}^{2}-2V_{1}\;\left|V_{2}\right|\;cos\left(\varphi \right)+{\left|V_{2}\right|}^{2}}$$

where *V*_1_ is the voltage measured by ADC1, *V*_2_ is the voltage measured by ADC2, *φ* is the phase of *V*_2_ and the phase of *V*_1_ is 0.

In order to reduce the noise of the measurement, the amplitudes and phases of the fundamental frequency were computed using the formula of Fourier transform. The result was then calculated by averaging the readings over 512 measurements and normalized by dividing every measurement by the maximum measured value.

The measuring circuit was calibrated by measuring commercially available capacitors and resistors and comparing the results with those of specialized instruments. For capacitance measurement calibration, an Extech LCR Meter (model 380193, Extech Instruments, Nashua, NH, USA) was used. For resistance measurements, a Keithley multimeter (Keithley 2000 Series, Keithley Instruments, Cleveland, OH, USA) was selected.

### 2.3. Mobile Application

**Figure 2:**
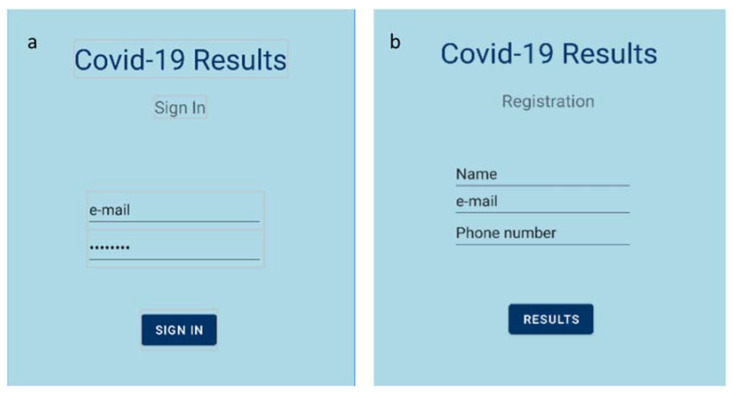
Mobile application’s homepage; (**a**) sign in page; (**b**) registration page.

In order to emphasize the main advantage of the developed sensor, i.e., the fast acquirement of the final result, an accompanying mobile application was developed, which is able to provide the test results in real-time. The developed application is based on the Bluetooth communication between an Android smartphone and the sensor’s board. The STM32 board lacks the ability to direct communication via Bluetooth. Hence, a transceiver module (HC-05), which is able to transmit data to the mobile application using the standard Bluetooth protocol, was added to the readout circuit. As a result, the Android application was able to display the detection of S protein in the tested sample in real time. A mockup of the developed application is illustrated in Figure 2.

Java was selected as the programming language of the mobile application. The development was performed using Google Android Studio [37]. The readout procedure is the following: The smartphone pairs with the Bluetooth device, i.e., the HC-05 module. Then, the device can be selected through the Android application, in order to establish a connection between the two parts. When the connection is established, the application receives the appropriate data packets in JSON format, sent from the readout circuit. All the necessary information is stored in those data packets, such as the outcome of the measurement, the measured value, and a timestamp. Finally, the test result (positive or negative) is displayed on the screen, after the user signs in to their personal account.

### 2.4. Swab Sample Collection and Biomedical Ethics Issues

All nasopharyngeal swab samples were collected from hospitalized patients by specialized personnel at the Konstantopoulio General Hospital (Athens, Greece), according to hospital safety standards. The medium used for the sample collection was the Citoswab transport medium VTM 3ml (product code 2118-0019). Regarding the collection, the Citoswab collection swab (product code 2122-0009, WellKang, Dover, UK) was used. The processing of the samples was performed in a class II biological safety cabinet using biosafety level three (BSL3) work practices. This research was conducted in such a way as to fully guarantee the patients’ anonymity and personal data confidentiality.

## 3. Results and Discussion

### 3.1. Readout Circuit Calibration

**Figure 3:**
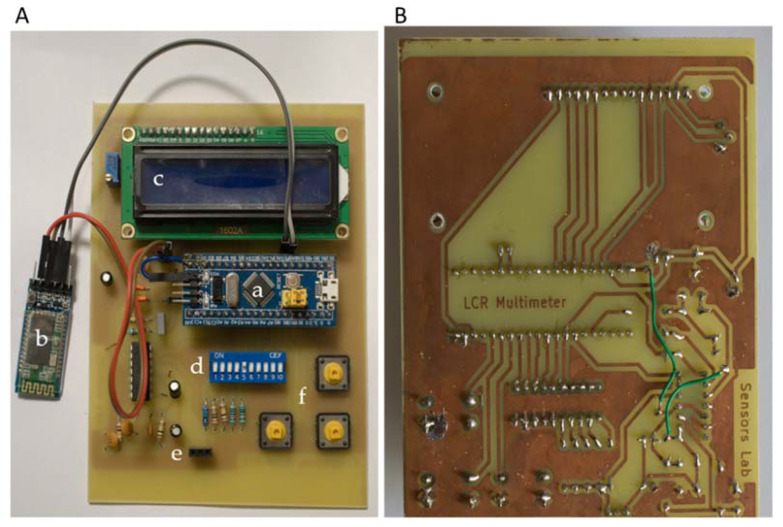
The prototype PCB; (**A**) front side of the board: (a) Blue Pill STM32 development board; (b) HC-05 BT module; (c) LCD screen; (d) DIP switches; (e) input pins; (f) settings buttons; (**B**) back side of the board.

The developed prototype board is illustrated in Figure 3. The front side of the board is illustrated in Figure 3A. In the center of the PCB, the Blue Pill STM32 development board was placed, along with the HC-05 BT module on the left, the LCD screen on top, and the DIP switches for selecting the suitable range below it. The device under test was connected on the left and right female pins of the three-pin connector at the bottom. Lastly, on the bottom right, there are three buttons responsible for specifying frequency and current range and for performing open-circuit calibration. The back side of the board is illustrated in Figure 3B. The total cost of the readout circuit was less than USD 20 (MCU (USD 7.5) + Bluetooth module (USD 6.5) + PCB, electronic components, case (USD ~5)), which makes it extremely competitive in price.

**Figure 4:**
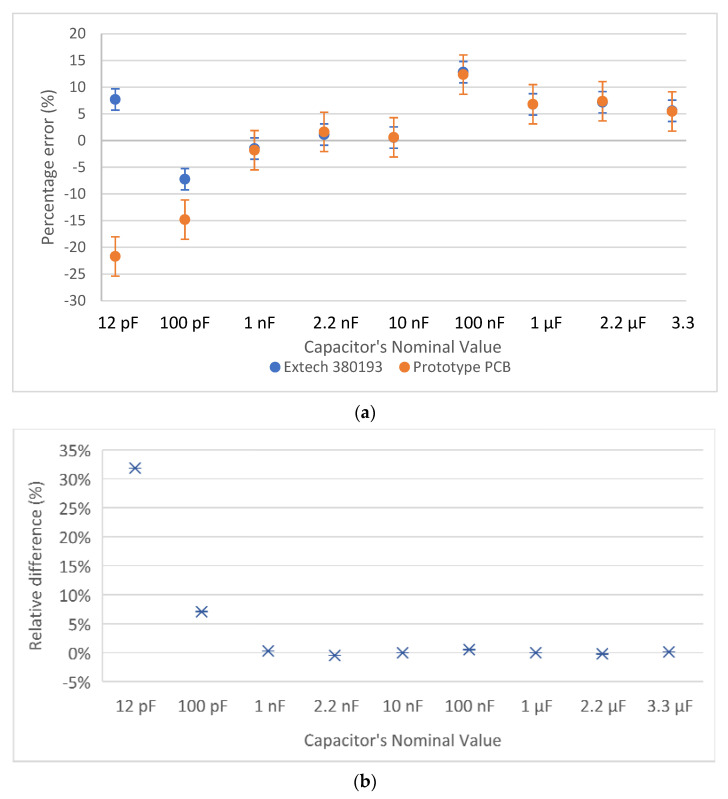
Capacitance measurements of 9 capacitors; (**a**) the absolute percentage difference between the nominal capacitance value, an LCR meter, and the developed circuit; (**b**) the relative difference between the developed circuit and the reference LCR meter.

**Figure 5:**
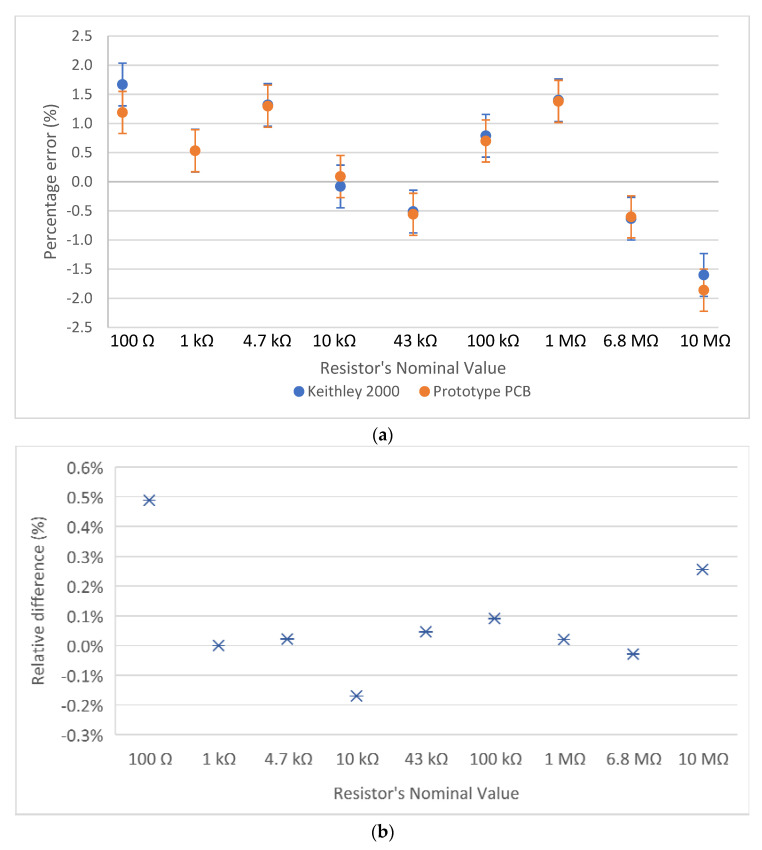
Resistance measurements of 9 resistors; (**a**) the absolute percentage difference between the nominal resistance value, a benchtop multimeter, and the developed circuit; (**b**) the relative difference between the developed circuit and the reference multimeter.

Capacitance measurement results are shown in Figure 4, and resistance measurement results are shown in Figure 5. More specifically, Figure 4a shows the percentage error that the measuring circuit and the Extech LCR meter exhibited during the capacitance measurements of nine different capacitors, having nominal capacitance values of 10 pF, 100 pF, 1 nF, 2.2 nF, 10 nF, 100 nF, 1 μF, 2.2 μF, and 3.3 μF. Figure 4b focuses on the relative difference between the measurements of the reference device and the measuring circuit, regarding the same nine capacitors.

Similarly, Figure 5a illustrates the percentage error that the measuring circuit and the Keithley multimeter exhibited while measuring the resistance of nine resistors, having nominal values of 100 Ω, 1 kΩ, 4.7 kΩ, 10 kΩ, 43 kΩ, 100 kΩ, 1 MΩ, 6.8 MΩ, and 10 MΩ. Figure 5b shows the relative difference between the measurements of the multimeter and the measuring circuit.

It is shown that the developed circuit can measure capacitance and resistance with high accuracy, across the desired range. The values measured by the developed circuit are very close to the values measured by the two reference instruments. The largest deviation between the measurements of the reference instrument and the developed circuit occurs for the 10 pF capacitor. However, it is possible that the error was due to the LCR meter, as the circuit’s measurement was closer to the nominal value. For every capacitor or resistor nominal value, 10 independent measurements were performed. The measurements shown in Figure 4 and Figure 5 correspond to the average value of these 10 measurements, whereas the error bars correspond to the standard deviation.

As has been pointed out in the introduction, integrating a biosensor into an electronic circuit for PoC treatment is a process that involves several challenges [27]. The main challenges we faced in this study were the stability and repeatability of the measurements. As is well known, a major challenge in the development of capacitive biosensors and especially in the design of their readout circuits is the treatment of noise interference [38,39,40]. In this case, the first idea was to measure the maximum, the minimum, and the time difference between the two maxima. This was the first method implemented. The disadvantage of the method, however, was that the signals had enough noise (ripple noise due to the fact that they are produced by using the sinusoidal pulse-width modulation (SPWM) technique, as well as electrical noise). As a result, the measurements exhibited a large dispersion. To address these issues, the amplitudes and phases of the fundamental frequency were computed using the formula of discrete Fourier transform. The result was then calculated by averaging the readings over 512 measurements and normalized by dividing every measurement by the maximum measured value.

### 3.2. Device Operation with Biological Fluids

**Figure 6:**
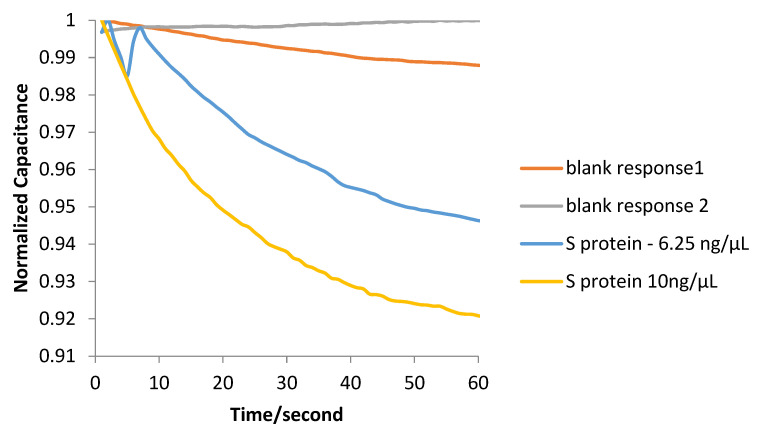
Normalized capacitance change over time for S protein.

**Figure 7:**
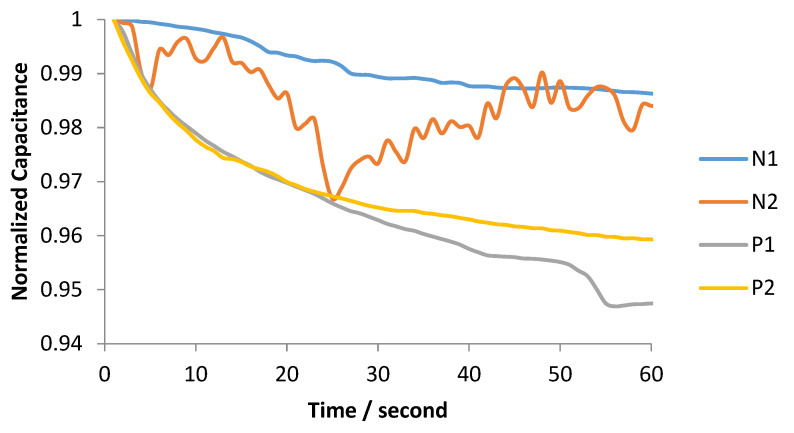
Normalized capacitance change over time for 4 swab samples, 2 negative to the virus (N1, N2) and 2 positive to the virus (P1, P2).

Experiments were conducted, both with solutions containing S protein and swab samples from hospitalized patients. Initially, four biosensors were prepared and kept at room temperature. At the surface of the first two, a blank solution containing only phosphate-buffered saline (PBS) was placed, in order to calculate the blank solution response, using an Eppendorf Research^®^ plus pipette. A 20 μL solution containing S protein (6.25 ng/μL) in phosphate-buffered saline (PBS) was placed on top of the third sensor and a 20 μL solution containing S protein (10 ng/μL) in PBS was placed on top of the fourth sensor. The effective capacitance change over time was monitored, as illustrated in Figure 6. Subsequently, experiments were conducted, targeting the detection of real virus molecules, acquired from swab samples of hospitalized patients. Four additional biosensors were prepared. At the surface of the first two sensors (N1, N2), only the Citoswab transport medium was placed, in order to calculate the blank solution response. At the surface of the other 2 (P1, P2), a 20μL solution of the Citoswab transport medium containing swab samples acquired from patients that were diagnosed positive for the virus was placed. The effective capacitance change over time was monitored, as illustrated in Figure 7.

The purpose of S protein measurements was to demonstrate that the original electronic circuit could satisfactorily measure protein S in biological samples and to separate samples containing protein S from those without it. As shown in Figure 6, for samples that do not contain protein S only a small change in capacitance is observed, whether it is a small increase or a small decrease. The two negative samples shown were selected as they relate to standard responses for samples that do not contain the protein. In contrast, in the case of samples containing protein S, a significant reduction in capacitance is observed, which, in fact, is proportional to the concentration of protein in the sample. Capacitance reduction is related to the displacement of the counter ions because of S protein binding on ACE2. Before placing the liquid sample in the biosensor, an ACE2 receptor layer has been immobilized on the gold electrode surface. Therefore, when a SARS-CoV-2 particle or S protein molecule binds to the ACE2 layer, a displacement of the counter ions around the capacitive electrode results in a decrease in its effective capacitance [41]. The higher the number of virus molecules bound to ACE2, the greater the decrease in the transducer’s capacitance (and therefore the change of the total impedance), detected as an electric signal [28].

As for the measurements with real virus samples shown in Figure 7, it was selected again to illustrate the response for two samples that were negative for the virus (N1, N2) and two samples that were positive for the virus (P1, P2). The positive samples were found positive for the virus after being tested with the real-time PCR method, with CT (cycle threshold) equal to 22 ± 0.2 (P1) and 26 ± 0.1 (P2). Out of the negative samples, N1 was selected to be illustrated, as its response is the one closer to the typical response of the negative samples. N2 refers to a single measurement and it was selected to be shown as an extreme case, with intense noise interference. Such a signal response was noticed only in one out of the sixteen negative samples that were tested. Malfunctions like this one that could be related either to a mistake during the biosensor development or, most probably, to a bad connection of the biosensor to the readout device, are a priority to address in our future work. In any case, the purpose of Figure 7 is to prove that positive samples can be distinguished from negative samples, by the fact that their measured response is a decreasing curve, and their maximum capacitance change is more than 2%. Reproducibility of the experiments, both with S protein and real virus samples, was demonstrated with three replicates. The capacitance values shown in Figure 6 and Figure 7 refer to the average value of the three experiments with the same sample, except for the measurement of the N2 sample, which refers to a single measurement. All experiments were performed at room temperature.

### 3.3. Measurement Procedure and Wireless Transmission to Mobile Application

**Figure 8:**
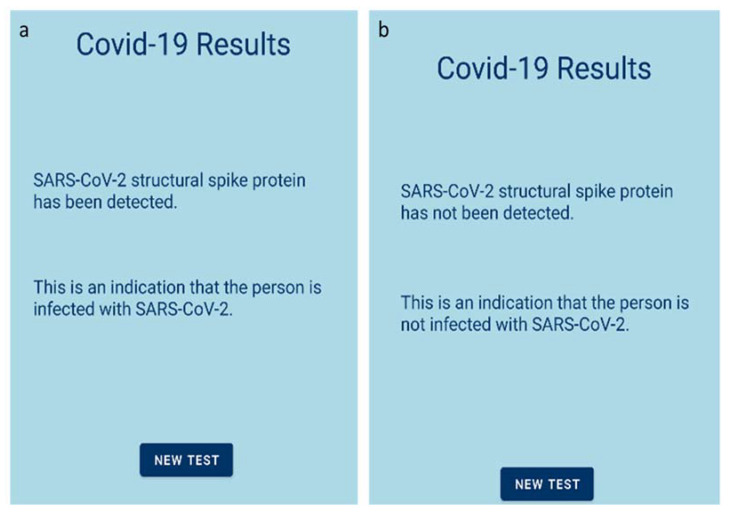
Mobile application results; (**a**) test positive for S protein; (**b**) test negative for S protein.

The measurement procedure is the following: The user has to open the Android application and register by entering some personal information (Figure 2b) or sign in if the user has already been registered, as shown in Figure 2a. Then, the testing procedure begins. After 60 s of measuring with a rate of one measurement per second, the resulting value is transmitted to the Android application via Bluetooth. If (a) the capacitance response was decreasing and (b) the total capacitance change exceeds 2%, the test is listed as positive for the SARS-CoV-2 S protein and the user receives the appropriate response (Figure 8a). Otherwise, if capacitance was not decreasing or the total capacitance change was below 2%, the test is listed as negative, and the user is informed as well (Figure 8b).

As stated in the introduction section, part of the purpose of a PoC device is to be friendly to use. The development of the mobile application fulfills that purpose, as it is much easier for the user to connect to its mobile phone and read if s/he is positive or negative to the virus than having to conclude that after reading continuous capacitance measurements.

However, it is not only the friendly to use concept that we achieve. The main advantages of this device are the simplicity in the construction and use, the portability, and the possibility that offers regarding the collection and processing of the test results. The latter advantage is particularly important, as a good knowledge of the distribution of cases geographically and temporally is a prerequisite for a successful pandemic policy. The wireless transmission to the mobile application is actually a single example of the possibilities that could be achieved when integrating the biosensor-based screening tests into the Internet of Things.

Regarding the screening tests, when used as diagnosing tools for a small number of patients, they may not have the efficiency and sensitivity of other methods, such as real-time PCR, which we do not attempt to replace in this research. Real-time PCR remains the “golden standard” for SARS-CoV-2 testing; however, it is not a screening device focused on PoC treatment like the one we demonstrate in this work. Screening tests are usually more effective when used in large sections of the population or population groups. Therefore, it is important for screening tests to be designed in a way that is easily acceptable to people and it is important for the test results to be easily assembled and processed, something that is made possible by the IoT and the biosensor platform presented.

Regarding the calibration of the device, it has been taken into account that it is a device intended for medical screening, in which the non-existence of false positives of results is considered a priority, in order to avoid initiating harmful diagnostic testing, and squandering health-care resources [42]. That is why, even though the maximum capacitance decrease caused by the negative samples was lower than 2%, it was selected to list all the results with a capacitance decrease of 2% or less as negative, in order to avoid having false positive results.

## 4. Conclusions

In this project, a SARS-CoV-2 S protein detecting device was developed using the ACE2-based capacitance sensor for rapid native SARS-CoV-2 detection [10]. The device consists of a microcontroller-based electronic circuit that, as shown, can measure capacitance and resistance change with high accuracy, and an Android application, where the test results are transmitted via Bluetooth. In this paper, new experiments were conducted targeting directly SARS-CoV-2 particles in swab samples of hospitalized patients. The device proved to be able to accurately measure the change in capacitance, both for protein S and for swab samples containing virus particles. Regarding the calibration of the device, it was observed that the noise introduced by the Citoswab transport medium can be significant and therefore the conditions for a sample to be considered positive for the virus were modified to: (a) “the capacitance response is constantly decreasing” and (b) “the total capacitance change exceeds 2%”.

## References

[1] Morales-Narváez E., Dincer C. (2020). The impact of biosensing in a pandemic outbreak: COVID-19. Biosens. Bioelectron..

[2] Allan-Blitz L.T., Klausner J.D. (2021). A Real-World Comparison of SARS-CoV-2 Rapid Antigen Testing versus PCR Testing in Florida. J. Clin. Microbiol..

[3] Kohmer N., Toptan T., Pallas C., Karaca O., Pfeiffer A., Westhaus S., Widera M., Berger A., Hoehl S., Kammel M. (2021). Article the comparative clinical performance of four SARS-CoV-2 rapid antigen tests and their correlation to infectivity in vitro. J. Clin. Med..

[4] Scohy A., Anantharajah A., Bodéus M., Kabamba-Mukadi B., Verroken A., Rodriguez-Villalobos H. (2020). Low performance of rapid antigen detection test as frontline testing for COVID-19 diagnosis. J. Clin. Virol..

[5] Eshghifar N., Busheri A., Shrestha R., Beqaj S. (2021). Evaluation of Analytical Performance of Seven Rapid Antigen Detection Kits for Detection of SARS-CoV-2 Virus. Int. J. Gen. Med..

[6] Li R., Liu J., Zhang H. (2021). The challenge of emerging SARS-CoV-2 mutants to vaccine development. J. Genet. Genom..

[7] Imran S., Ahmadi S., Kerman K. (2021). Electrochemical biosensors for the detection of SARS-CoV-2 and other viruses. Micromachines.

[8] Abid S.A., Ahmed Muneer A., Al-Kadmy I.M.S., Sattar A.A., Beshbishy A.M., Batiha G.E.S., Hetta H.F. (2021). Biosensors as a future diagnostic approach for COVID-19. Life Sci..

[9] Mahmoudinobar F., Britton D., Montclare J.K. (2021). Protein-based lateral flow assays for COVID-19 detection. Protein Eng. Des. Sel..

[10] Tahamtan A., Ardebili A. (2020). Real-time RT-PCR in COVID-19 detection: Issues affecting the results. Expert Rev. Mol. Diagn..

[11] Rasmi Y., Li X., Khan J., Ozer T., Choi J.R. (2021). Emerging point-of-care biosensors for rapid diagnosis of COVID-19: Current progress, challenges, and future prospects. Anal. Bioanal. Chem..

[12] Shen Y., Anwar T.B., Mulchandani A. (2021). Current status, advances, challenges and perspectives on biosensors for COVID-19 diagnosis in resource-limited settings. Sens. Actuators Rep..

[13] Iliescu F.S., Ionescu A.M., Gogianu L., Simion M., Dediu V., Chifiriuc M.C., Pircalabioru G.G., Iliescu C. (2021). Point-of-care testing-the key in the battle against SARS-CoV-2 pandemic. Micromachines.

[14] Mehrdad S., Wang Y., Atashzar S.F. (2021). Perspective: Wearable Internet of Medical Things for Remote Tracking of Symptoms, Prediction of Health Anomalies, Implementation of Preventative Measures, and Control of Virus Spread During the Era of COVID-19. Front. Robot. AI.

[15] Chandra M., Kumar K., Thakur P., Chattopadhyaya S., Alam F., Kumar S. (2022). Digital technologies, healthcare and COVID-19: Insights from developing and emerging nations. Health Technol..

[16] Malliga S., Kogilavani S.V., Nandhini P.S. (2021). A Comprehensive Review of Applications of Internet of Things for COVID-19 Pandemic. IOP Conf. Ser. Mater. Sci. Eng..

[17] Mavrikou S., Tsekouras V., Hatziagapiou K., Paradeisi F., Bakakos P., Michos A., Koutsoukou A., Konstantellou E., Lambrou G.I., Koniari E. (2021). Clinical Application of the Novel Cell-Based Biosensor for the Ultra-Rapid Detection of the SARS-CoV-2 S1 Spike Protein Antigen: A Practical Approach. Biosensors.

[18] Fathi-Hafshejani P., Azam N., Wang L., Kuroda M.A., Hamilton M.C., Hasim S., Mahjouri-Samani M. (2021). Two-Dimensional-Material-Based Field-Effect Transistor Biosensor for Detecting COVID-19 Virus (SARS-CoV-2). ACS Nano.

[19] Rashed M.Z., Kopechek J.A., Priddy M.C., Hamorsky K.T., Palmer K.E., Mittal N., Valdez J., Flynn J., Williams S.J. (2021). Rapid detection of SARS-CoV-2 antibodies using electrochemical impedance-based detector. Biosens. Bioelectron..

[20] Seo G., Lee G., Kim M.J., Baek S.H., Choi M., Ku K.B., Lee C.S., Jun S., Park D., Kim H.G. (2020). Rapid Detection of COVID-19 Causative Virus (SARS-CoV-2) in Human Nasopharyngeal Swab Specimens Using Field-Effect Transistor-Based Biosensor. ACS Nano.

[21] Garg M., Sharma A.L., Singh S. (2021). Advancement in biosensors for inflammatory biomarkers of SARS-CoV-2 during 2019–2020. Biosens. Bioelectron..

[22] Sharma P.K., Kim E.-S., Mishra S., Ganbold E., Seong R.-S., Kaushik A.K., Kim N.-Y. (2021). Ultrasensitive and Reusable Graphene Oxide-Modified Double-Interdigitated Capacitive (DIDC) Sensing Chip for Detecting SARS-CoV-2. ACS Sens..

[23] McCarthy M.W. (2021). At-home coronavirus testing: The next game-changer?. Expert Rev. Mol. Diagn..

[24] Toppings N.B., Mohon A.N., Lee Y., Kumar H., Lee D., Kapoor R., Singh G., Oberding L., Abdullah O., Kim K. (2021). A rapid near-patient detection system for SARS-CoV-2 using saliva. Sci. Rep..

[25] Donato L.J., Trivedi V.A., Stransky A.M., Misra A., Pritt B.S., Binnicker M.J., Karon B.S. (2021). Evaluation of the Cue Health point-of-care COVID-19 (SARS-CoV-2 nucleic acid amplification) test at a community drive through collection center. Diagn. Microbiol. Infect. Dis..

[26] Ravi N., Cortade D.L., Ng E., Wang S.X. (2020). Diagnostics for SARS-CoV-2 detection: A comprehensive review of the FDA-EUA COVID-19 testing landscape. Biosens. Bioelectron..

[27] Singh A., Sharma A., Ahmed A., Sundramoorthy A.K., Furukawa H., Arya S., Khosla A. (2021). Recent advances in electrochemical biosensors: Applications, challenges, and future scope. Biosensors.

[28] Georgas A., Lampas E., Houhoula D.P., Skoufias A., Patsilinakos S., Tsafaridis I., Patrinos G.P., Adamopoulos N., Ferraro A., Hristoforou E. (2022). ACE2-based capacitance sensor for rapid native SARS-CoV-2 detection in biological fluids and its correlation with real-time PCR. Biosens. Bioelectron..

[29] Mazlan N.S., Ramli M.M., Abdullah M.M.A.B., Halin D.S.C., Isa S.S.M., Talip L.F.A., Danial N.S., Murad S.A.Z. (2017). Interdigitated electrodes as impedance and capacitance biosensors: A review. AIP Conf. Proc..

[30] Lan J., Ge J., Yu J., Shan S., Zhou H., Fan S., Zhang Q., Shi X., Wang Q., Zhang L. (2020). Structure of the SARS-CoV-2 spike receptor-binding domain bound to the ACE2 receptor. Nature.

[31] Shang J., Ye G., Shi K., Wan Y., Luo C., Aihara H., Geng Q., Auerbach A., Li F. (2020). Structural basis of receptor recognition by SARS-CoV-2. Nature.

[32] Vásquez V., Navas M.-C., Jaimes J.A., Orozco J. (2022). SARS-CoV-2 electrochemical immunosensor based on the spike-ACE2 complex. Anal. Chim. Acta.

[33] Suh J.-S., Kim H.-S., Kim T.-J. (2021). Development of a SARS-CoV-2-derived receptor-binding domain-based ACE2 biosensor. Sens. Actuators B Chem..

[34] Lee J.-H., Lee Y., Lee S.K., Kim J., Lee C.-S., Kim N.H., Kim H.G. (2022). Versatile role of ACE2-based biosensors for detection of SARS-CoV-2 variants and neutralizing antibodies. Biosens. Bioelectron..

[35] Georgas A., Agiannis K., Papakosta V., Angelopoulos S., Ferraro A., Hristoforu E. (2022). A portable screening device for SARS-CoV-2 with smartphone readout. Eng. Proc..

[36] Karki J. (2000). Active Low-Pass Filter Design.

[37] Cheon Y. Multiplatform application development for android and java. Proceedings of the 2019 IEEE 17th International Conference on Software Engineering Research, Management and Applications (SERA).

[38] Ng C.L., Reaz M.B.I., Chowdhury M.E.H. (2020). A Low Noise Capacitive Electromyography Monitoring System for Remote Healthcare Applications. IEEE Sens. J..

[39] Ng C.L., Reaz M.B.I. (2017). Characterization of textile-insulated capacitive biosensors. Sensors.

[40] Lee K.H., Choi S., Lee J.O., Yoon J.B., Cho G.H. CMOS capacitive biosensor with enhanced sensitivity for label-free DNA detection. Proceedings of the 2012 IEEE International Solid-State Circuits Conference.

[41] Mattiasson B., Hedström M. (2016). Capacitive biosensors for ultra-sensitive assays. TrAC—Trends Anal. Chem..

[42] Grimes D.A., Schulz K.F. (2002). Uses and abuses of screening tests. Lancet.

